# Predictors of Late Adverse Outcomes After Carotid Endarterectomy

**DOI:** 10.3390/medicina62030593

**Published:** 2026-03-21

**Authors:** Danka Vukasinovic, Milos Maksimovic, Slobodan Tanaskovic, Jelena Marinkovic, Andja Cirkovic, Branko Jakovljevic, Jelena Ilic Zivojinovic, Djordje Radak, Hristina Vlajinac

**Affiliations:** 1 Institute of Hygiene and Medical Ecology, Faculty of Medicine, University of Belgrade, 11000 Belgrade, Serbia; 2 Vascular Surgery Clinic, Dedinje Institute for Cardiovascular Diseases, 11000 Belgrade, Serbia; 3 Faculty of Medicine, University of Belgrade, 11000 Belgrade, Serbia; 4 Institute of Medical Statistics and Informatics, Faculty of Medicine, University of Belgrade, 11000 Belgrade, Serbia; 5 Department of Vascular Surgery, Serbian Academy of Art and Sciences, Euromedic Clinic, 11000 Belgrade, Serbia; 6 Insitute of Epidemiology, Faculty of Medicine, University of Belgrade, 11000 Belgrade, Serbia

**Keywords:** carotid endarterectomy, outcomes, predictors, myocardial infarction, stroke, death, restenosis

## Abstract

*Background and Objectives:* Although carotid endarterectomy (CEA) is the gold standard in the treatment of carotid disease, a higher frequency of adverse outcomes can reduce its benefit. The aim of the present study is to identify factors related to myocardial infarction, stroke, death and restenosis as the late adverse outcomes of CEA. *Materials and Methods:* The retrospective cohort study included 1597 CEAs that were performed in 1533 consecutive patients at the Vascular Surgery Clinic in Belgrade from 2012 to 2017. Late adverse outcomes within 4 years after CEA were available for the majority of them. Data for myocardial infarction and stroke were available for 1223 CEAs, data for death for 1305 CEAs, and data for restenosis for 1162 CEAs. The association between possible risk factors and late adverse outcomes of CEA was analyzed using univariate and multivariate Cox and logistic regression analyses. *Results:* During follow-up, myocardial infarction occurred after 55, stroke after 68, death after 103 and restenosis after 121 CEAs. Two factors were the most frequent predictors of late adverse outcomes, i.e., the patient’s age and diabetes mellitus (DM). Age predicted all late adverse outcomes except restenosis, and DM predicted all of them. A predictor of myocardial infarction, besides age (HR 1.08, 95% CI 1.05–1.11) and DM (HR 1.60, 95% CI 1.11–2.29), was peripheral arterial disease (HR 1.81, 95% CI 1.17–2.78) in personal history. Predictors were only age (HR 1.04, 95% CI 1.01–1.08) and DM (HR 1.68, 95% CI 1.03–2.72) for stroke, as well as for death (HR 1.17, 95% CI 1.12–1.21 and HR 1.94, 95% CI 1.17–3.21, respectively). For restenosis, in addition to DM (HR 1.78, 95% CI 2.62), predictors were hyperlipidemia (HR 3.52, 95% CI 1.27–9.76) and urgent surgery (HR 3.51, 95% CI 1.06–11.65). *Conclusions:* CEA should be performed with special caution in the elderly and diabetic patients. Modification of other risk factors and precise medical therapy are necessary to reduce possible adverse outcomes.

## 1. Introduction

Carotid endarterectomy (CEA) is considered the gold standard in the treatment of extracranial carotid disease [1]. Although the goal of CEA is long-term stroke prevention, sometimes it can be followed by early and late adverse outcomes. Late adverse outcomes of CEA are the measure of its efficiency, and a higher frequency of them can reduce its benefit, particularly in asymptomatic patients [2]. Identifying patients with an increased risk of postoperative complications would enable adequate treatment selection. Besides carotid artery stenosis, these patients often have atherosclerotic plaque depositions in other vascular territories due to the systemic nature of atherosclerosis. This atherosclerotic burden, even if asymptomatic at the time of CEA, could cause major cardiovascular events in the future. Therefore, risk factor management plays a key role in preventing future adverse outcomes.

Many studies investigated possible predictors of late adverse outcomes of CEA, but their results were inconsistent [3,4,5,6]. Predictors have varied depending on the specific outcome that was monitored, duration of the follow-up period, or preoperative variables included in the analyses. In any case, their identification should influence a more detailed clinical assessment of patients, attempting to reduce significant risk factors before surgery, as well as more frequent control examinations of those patients after surgery and a more precise selection of an appropriate postoperative therapy, all in order to improve the prognosis of CEA.

The aim of the present study is to find out what factors are related to myocardial infarction, stroke, death and restenosis as the late adverse outcomes of CEA.

## 2. Materials and Methods

The retrospective cohort study included 1597 CEAs that were performed in 1533 consecutive patients at the Clinic for Vascular Surgery of the Institute for Cardiovascular Diseases “Dedinje” in Belgrade, from 1 January 2012 to 31 December 2017. Myocardial infarction, stroke, death and restenosis after 30 postoperative days and within 4 years after CEA were considered late adverse outcomes. The diagnosis of myocardial infarction was established in patients with an increase in cardio-specific enzymes and electrocardiographic or echocardiographic changes. Stroke was defined as any stroke. Restenosis was defined as the development of >50% stenosis of the operated carotid artery, according to the North American Symptomatic Carotid Endarterectomy Trial (NASCET) criteria. Death referred to all-cause mortality. But during follow-up, data related to the late adverse outcomes were not available for all of them. Data for myocardial infarction and stroke were available for 1223 CEAs, data for death for 1305 CEAs, and data for restenosis for 1162 CEAs. Furthermore, we excluded patients who had CEA and simultaneous coronary artery bypass grafting, patients who previously had massive cerebrovascular insult (CVI) and severe neurological damage (infarction zone > 1/3 middle cerebri artery (MCA) territory or modified Rankin Scale (mRS) ≥ 4), patients with severe renal insufficiency (estimated Glomerular Filtration Rate (eGFR) < 30 mil/min, chronic kidney disease stage 4 or 5), and patients with severe cardiac comorbidity (NYHA class 3 or 4, ejection fraction (EF) < 30%, unstable angina pectoris and recent myocardial infarction within 30 days prior CEA).

Data collected from the patients’ medical records included: sex, age, body height and body weight, smoking status, personal history and family history, levels of triglycerides and total cholesterol at admission, characteristics of carotid disease (symptomatic status, type of plaque and degree of carotid stenosis), operative data (type of surgery—urgent or elective—and clamp duration) and therapy used before CEA and after hospital discharge.

Eversion CEA was performed in all patients, and only 3.8% of patients underwent bilateral procedures. Urgent endarterectomy was performed in patients with crescendo TIA or stroke in evolution within 48 h of symptom onset [7]. After surgery, all patients were uniformly treated with best medical therapy (antiplatelet therapy and statins), with additional therapy in those with other comorbidities.

Patient follow-ups were four years after CEA. Clinical examinations and duplex scans were performed 1 year after operation and annually thereafter. When patients did not come for the control examination at the clinic where they were operated, they were interviewed by phone. The phone interview was conducted with the study participant or, sometimes, with a member of his/her family. During follow-up, data of patients’ survival and causes of death, instances of stroke and myocardial infarction and their time of occurrence, as well as data of restenosis were collected. Only the first event of each outcome was included in the analysis.

The study protocol was approved by the Ethics Committee of the Faculty of Medicine, University of Belgrade (No 1322/XII-1).

### Statistical Analysis

Categorical variables were presented as numbers and percentages and continuous variables as means and standard deviations. The association between possible risk factors and major late adverse outcomes, myocardial infarction, stroke and death were analyzed using univariate and multivariate Cox regression analyses. Predictors for restenosis, ever detected during follow-up, were analyzed using univariate and multivariate logistic regression analyses. Variables that, according to both univariate Cox regression and univariate logistic regression analysis, differed between compared groups at a level of *p* ≤ 0.10 were included in the multivariate analysis. The selection method was backward Wald. All *p* values are based on a two-tailed test, and *p* < 0.05 was considered significant. In order to assess the median time to event, Kaplan–Meier analysis was performed. To assess potential bias due to missing values, we performed multiple imputation of the dichotomous outcome variables (myocardial infarction, stroke, death, and restenosis). First, a pattern analysis was conducted, which showed that no variable suitable for imputation had more than 1% of missing values. Next, multiple imputation was performed. Five imputations were defined, and the fully conditional specification and Markov Chain Monte Carlo (FCS/MCMC) methods with a maximum of 20 iterations were also applied. All variables potentially relevant for the outcomes were included solely as predictors, while the outcome variables were just imputed. Afterword, Cox and logistic regression analysis were repeated in the same manner on imputed data reporting pooled HRs and 95% confidence intervals. Pooled results, according to Rubin`s rule of multiple imputed databases, for all outcomes are presented in Results section Statistical Package for Social Sciences (SPSS), version 30, was used for the analysis.

## 3. Results

The distribution of the observed characteristics of patients with and without late adverse outcomes after CEAs are presented in Table 1 and Table 2.

Myocardial infarction occurred after 55 (4.5%) CEAs out of 1223 procedures. According to univariate Cox regression analysis, it was more frequent among patients who were older, male, and had ACB, chronic heart failure, PAD and diabetes mellitus (DM) in their personal history. Stroke appeared after 68 (5.6%) of 1223 CEAs. Patients with stroke differed significantly, compared with those without stroke, in age (older), more frequent DM in their personal history and more frequent use of clopidogrel in their preoperative therapy. Those who died during four years of follow-up, 103 (7.9%) of 1305 CEAs, in comparison to survivors, were older, more frequently males, more frequently had DM, and more frequently used statins preoperatively. Restenosis occurred after 121 (10.4%) of 1162 CEAs. According to univariate regression analysis, restenosis was associated with hyperlipidemia, hypertension and DM (Table 1).

According to data presented in Table 2, patients with myocardial infarction (MI) more frequently used OACs in the hospital discharge therapy, compared with those without MI. Stroke was significantly related to less frequent use of aspirin in the hospital discharge therapy. Death was associated with more frequent use of OACs in the hospital discharge therapy, and urgent CEA was more frequent in those with restenosis.

Results of crude, adjusted, and multiple imputed regression models for myocardial infarction, stroke, death and restenosis, as the late adverse outcomes of CEAs, are presented in Table 3. Two factors were the most frequent predictors of late adverse outcomes: the patient’s age and the presence of DM. DM was the predictor for all late adverse outcomes, and concurrent patients’ age was not predictor only for restenosis. For myocardial infarction, besides age (HR 1.08, 95% CI 1.05–1.11) and DM (HR 1.60, 95% CI 1.11–2.29), predictor was PAD in the personal history (HR 1.81, 95% CI 1.17–2.78). Previous ACB was a predictor for myocardial infarction in the unadjusted model (HR 1.70, 95% CI 1.02–2.84), but after adjustment and multiple imputation, its statistical significance was lost. Also, the association of OACs in the hospital discharged therapy with myocardial infarction became significant only in the multiple imputed regression model (HR 1.97, 95% CI 1.02–3.80). Predictors of stroke were age (HR 1.04, 95% CI 1.01–1.08) and DM (HR 1.68, 95% CI 1.03–2.72). Also, age (HR 1.17, 95% CI 1.12–1.21) and DM (HR 1.94, 95% CI 1.17–3.21), were the predictors for death. OACs in the hospital discharge therapy were associated with death in crude and imputed models. But in the adjusted model for all preoperative therapy and cardiovascular comorbidities, the predictive value of OACs was lost. For restenosis, in addition to DM, the predictors were hyperlipidemia (HR 3.52, 95% CI 1.27–9.76) and urgent surgery (HR 3.51, 95% CI 1.06–11.65). The association of urgent surgery with restenosis remained after adjustment but was not confirmed after multiple imputation.

When restenosis was added in the new multivariate analyses, it was found to be a significant predictor for stroke (HR = 3.63; 95% CI 1.91–6.91; *p* < 0.001) and for late major adverse outcomes (MAOs) that is, for myocardial infarction, stroke and death taken together (HR = 2.50, 95% CI 1.37–4.54).

Out of 1162 CEAs, stroke occurred in 46. Restenosis was present in 13 of them (28.3%) and in 33 (3.0%) of those without stroke. Among those with late MAOs, restenosis was present in 14, and 13 of them were stroke related.

**Figure 1:**
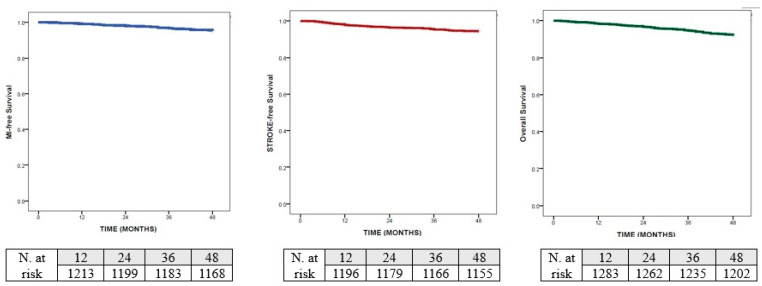
Kaplan–Meier analysis for: myocardial infarction; stroke and death.

According to the Kaplan–Meier analysis, the median time to myocardial infarction was not reached, but the cumulative incidence at 48 months was 4.5% and the restricted mean survival time to acute myocardial infarction was 47.05 months (95% CI 46.76–47.34). Similarly, the cumulative incidence for stroke at 48 months was 5.6% and the restricted mean survival time to stroke was 46.47 months (95% CI 46.09–46.88). Finally, the cumulative incidence of death at 48 months was the highest (7.9%) and the restricted mean survival time to death was 46.35 months (95% CI 45.97–46.72)—Figure 1.

## 4. Discussion

The present study demonstrates that the presence of DM and patients’ age are the main predictors for the late adverse outcomes of CEA. DM was a significant predictor of all late adverse outcomes, whereas age was a significant predictor of all of them, except restenosis. All other factors, predictors for late adverse outcomes were, significantly and independently from each other, related to only one of late complications.

Age and DM are among main risk factors for the development of cardiovascular diseases and consequently, for death [8,9,10]. In our study, in agreement with others [11,12,13,14,15], age was identified as a predictor of shorter long-term survival after CEA. Also, we identified age as an independent risk factor for two other late major adverse outcomes, such as stroke and myocardial infarction. Similar to our results, advanced age was a predictor of long-term overall stroke [14], or the combined endpoint of any stroke/death during follow-up [16]. On the contrary, a recent study [17] found no difference between octogenarians and younger patients who had undergone CEA, regarding both early and late endpoints.

The prevalence of DM is high among patients undergoing carotid endarterectomy [1]. In the majority of studies, DM influenced poor CEA outcomes, but there have also been studies in which no difference was observed between patients with and without DM in the frequency of adverse outcomes after CEA [1,18,19,20,21,22,23]. In some studies, the relationship between DM and complications after CEA differed depending on whether patients were symptomatic or asymptomatic [20], or depending on whether complications were early or late [18,21]. In our study, DM was independent risk factor for all late adverse outcomes. According to the results of many studies [13,15,21,24,25,26], DM increases the risk for death during follow-up, especially cardiac-related death [24,26]. In meta-analysis from 2016, Hussain et al. concluded that diabetic patients were at an increased long-term risk for death after both carotid revascularization procedures, endarterectomy and stenting [27]. A recent Swedish study showed that patients with DM had a 30% higher long-term risk for stroke after CEA than patients without DM [28]. In the same study, patients with DM had a higher risk for death, as well as major adverse outcomes (myocardial infarction, stroke or death). Another study by the same authors, has revealed that poor glycemic control (assessed based on HbA1C values) is associated with a higher long-term risk of stroke or death [29]. Jeong et al. identified DM as a predictor of long-term stroke after CEA [21]. In this study, stroke rates were 7.2% in patients with DM, and 2.7% in patients without DM, confirming that the efficiency of CEA as a long-term stroke prevention procedure was worse in the presence of DM [21]. In our study, DM was also associated with an increased risk of restenosis after CEA [1,18,30,31,32]. Earlier studies explained that DM may increase neointimal hyperplasia and accelerate the growth of new atherosclerotic plaque at the site of arterial injury, referring to the carotid artery as well [1,32,33].

Besides age and DM, we found that the predictor for late myocardial infarction after CEA was PAD in the personal history. PAD in the personal history reflects a systemic nature of atherosclerosis and often coexists with carotid disease. In the study by van Lammeren et al. [34], the history of PAD was one of the most important factors of developing major cardiovascular events after CEA. Similar, Ogata et al. [35] found that previous myocardial infarction or PAD were strongly associated with future occurrences of myocardial infarction. The independent effect of PAD on predicting major cardiovascular events during long-term follow-up after CEA was also confirmed in the study by Vilarino-Rico et al. [4].

In our study, previous ACB emerged as a predictor of late myocardial infarction in the unadjusted analysis. However, this association disappeared after adjustment for cardiovascular comorbidities and preoperative therapy. This suggests that ACB likely reflects a higher baseline cardiovascular risk profile rather than acting as an independent predictor of myocardial infarction during follow-up. Importantly, the absence of an independent association persisted after applying multiple imputation, indicating that the result was not driven by missing data.

OACs in the hospital discharge therapy was not associated with myocardial infarction during follow-up, both in the unadjusted and adjusted models. But this association became statistically significant after multiple imputation. Similarly, we found an association between the use of OACs after hospital discharge and death during follow-up. These associations suggest that the complete-case analysis may have been affected by missing data and reduced statistical power. This influence of missing data is omitted now.

In the present study, in addition to DM, hyperlipidemia was identified as a predictor of restenosis after CEA. Dyslipidemia is a well-established risk factor for atherosclerotic disease. Several studies have reported that dyslipidemia is a risk factor for restenosis after carotid revascularization procedures [30,36,37,38]. The pathophysiologic mechanism involves vascular damage by oxidation of low-density lipoprotein and formation of unstable, foamy, necrotic, atherosclerotic carotid plaques [39]. In meta-analysis from 2019, Texakalidis et al. showed that dyslipidemia was associated with restenosis after carotid revascularization procedures CEA and CAS when they were considered together but not after CEA alone [30].

Urgent endarterectomy (≤48 h) was associated with restenosis in the complete-case analysis, both unadjusted and adjusted. In the literature, urgent endarterectomy (≤48 h) was reported as a risk factor for perioperative outcomes [40,41,42,43], particularly stroke. Regarding late outcomes, some authors did not find a difference in the long-term risk of stroke or death between urgent and elective CEA [43,44], but there is a lack of studies on its impact on restenosis. However, in our study, this association was not confirmed after applying multiple imputation, suggesting that the initial finding may not be robust. Patients undergoing urgent intervention frequently present with symptomatic and potentially more unstable disease, which may reflect a higher baseline risk profile than a direct effect of surgical urgency itself. In addition, the loss of the predictive value of urgent surgery after multiple imputation may be due to the very small number of urgent CEAs included in the complete-case analysis (only 14).

When we added restenosis in the new Cox proportional hazard model, it was found to be the predictor for long-term stroke, similar to the results of other studies [30,45,46]. Secondary analysis of the International Carotid Stenting Study (ICSS) showed that patients with at least moderate restenosis (≥50%) had a higher risk of ipsilateral stroke in the overall study group (patients with CEA and CAS) and in the CEA group alone but no significant increase in stroke risk after restenosis was recorded in the CAS group [45]. The meta-analysis of nine RCTs found that CEA patients with an untreated, asymptomatic > 70% restenosis had a significantly higher risk of late ipsilateral stroke [46]. However, in our study, the effect of higher restenosis (>70%) was not examined. Also, we observed total long-term stroke, and not an ipsilateral one.

In our study, patient survival time, as well as event free survival during follow-up was quite long, almost 4 years. A previous study defined the risk of myocardial infarction, stroke or death after CEA as 6–35% at 3-years follow-up, depending on the baseline risk profiles of patients [34]. In the study by Go et al., a 5-year risk of stroke was 16% [3]. In our study, the cumulative incidence for stroke at 48 months was 5.6%, in accordance with the ultimate goal of CEA as a long-term stroke prevention procedure. The cumulative incidences at 48 months for myocardial infarction and death were 4.5% and 7.9%, respectively. Recent vascular guidelines emphasize the decline of these rates in best medical treatment (BMT) patients [47].

The present study has some limitations. First is the number of followed-up patients. Patients were lost to follow-up for different reasons: some of them died, some did not come to the control examinations, and some we could not contact at all. When we compared all baseline characteristics of followed and non-followed patients, the only significant difference between these groups was in age (Supplements Tables S1–S3). Second, patients’ adherence to the prescribed medications was not supervised, as well as medical therapy modification over follow-up period. Third, some relevant data (values of low-density and high-density lipoproteins, some procedural data and some comorbidities) were not included in the study as they were not available for all patients. Fourth, restenosis time was unavailable. Fifth, the single-center retrospective design of the study might limit the generalizability of our results, thus our findings should be considered with caution. However, our study is the first of its kind in Serbia.

## 5. Conclusions

To increase the efficiency of CEA for a long time, it is preferable to identify the risk factors for long-term adverse outcomes. Patients who undergo CEA often have other comorbidities that could result in MI, stroke, restenosis or death during long-term follow-up. Risk-factor modification and precise best medical therapy are very important for the reduction in future adverse outcomes after CEA. Due to the observational study design, our findings cannot prove causation, and future studies about the subject are necessary.

## Figures and Tables

**Table 1:** Demographic characteristics, BMI, smoking, personal history, therapy, laboratory values on admission and family history of cardiovascular diseases according to the occurrence of myocardial infarction, stroke, death and restenosis as the late adverse outcomes of carotid endarterectomy.

Variable	Myocardial Infarction *		Stroke *		Death *		Restenosis **	
	NO (n = 1168)	YES (n = 55)	NO (n = 1155)	YES (n = 68)	NO (n = 1202)	YES (n = 103)	NO (n = 1041)	YES (n = 121)
Age, y, mean ± SD	67.76 ± 7.62	**73.53 ± 7.83 ^a^**	67.88 ± 7.65	**70.37 ± 8.52 ^b^**	67.73 ± 7.61	**74.17 ± 7.37 ^a^**	67.63 ± 7.58	67.45 ± 7.38
Gender—Male, n (%)	685 (58.6)	**41 (74.5) ^c^**	685 (59.3)	41 (60.3)	706 (58.7)	**74 (71.8) ^c^**	613 (58.9)	69 (57.0)
BMI: underweight normal weight overweight obese	6 (0.5) 397 (34.0) 556 (47.6) 209 (17.9)	1 (1.8) 16 (29.1) 27 (49.1) 11 (20.0)	7 (0.6) 390 (33.8) 551 (47.7) 207 (17.9)	0 (0.0) 23 (33.8) 32 (47.1) 13 (19.1)	7 (0.6) 409 (34.0) 572 (47.6) 214 (17.8)	2 (1.9) 36 (35.0) 46 (44.7) 19 (18.4)	6 (0.6) 353 (33.9) 498 (47.8) 184 (17.7)	0 (0.0) 42 (34.7) 55 (45.5) 24 (19.8)
Smoking, n (%)	552 (47.3)	20 (36.4)	541 (46.8)	31 (45.6)	569 (47.3)	42 (40.8)	489 (47.0)	61 (50.4)
Personal history, n (%):								
Myocardial infarction	81 (6.9)	7 (12.7)	82 (7.1)	6 (8.8)	82 (6.8)	11 (10.7)	73 (7.0)	8 (6.6)
PCI	74 (6.3)	4 (7.3)	72 (6.2)	6 (8.8)	76 (6.3)	8 (7.8)	63 (6.1)	10 (8.3)
ACB	88 (7.5)	**13 (23.6) ^a^**	97 (8.4)	4 (5.9)	91 (7.6)	10 (9.7)	84 (8.1)	7 (5.8)
Chronic heart failure	16 (1.4)	**5 (9.1) ^a^**	20 (1.7)	1 (1.5)	18 (1.5)	3 (2.9)	17 (1.6)	1 (0.8)
Peripheral arterial disease	177 (15.1)	**14 (25.5) ^c^**	177 (15.3)	14 (20.6)	186 (15.5)	22 (21.4)	156 (15.0)	22 (18.2)
Aneurysmatic disease	37 (3.2)	3 (5.4)	38 (3.3)	2 (2.9)	39 (3.2)	5 (4.9)	33 (3.2)	5 (4.1)
Hyperlipidemia	1049 (89.8)	49 (89.1)	1037 (89.8)	61 (89.7)	1080 (89.9)	93 (90.3)	925 (88.9)	**117 (96.7) ^c^**
Hypertension	1094 (93.7)	51 (92.7)	1081 (93.6)	64 (94.1)	1126 (93.7)	96 (93.2)	970 (93.2)	**118 (97.5) ^d^**
Diabetes mellitus	349 (29.9)	**23 (41.8) ^d^**	344 (29.8)	**28 (41.2) ^c^**	356 (29.6)	**45 (43.7) ^b^**	293 (28.1)	**51 (42.1) ^b^**
Therapy before admission, n (%):								
Aspirin	962 (82.4)	44 (80.0)	947 (82.0)	59 (86.8)	988 (82.2)	83 (80.6)	849 (81.6)	106 (87.6)
Clopidogrel	321 (27.5)	14 (25.5)	309 (26.8)	**26 (38.2) ^c^**	324 (27.0)	31 (30.1)	282 (27.1)	32 (26.4)
OACs	53 (4.5)	5 (9.1)	55 (4.8)	3 (4.4)	54 (4.5)	7 (6.8)	49 (4.7)	4 (3.3)
ACEIs	849 (72.7)	44 (80.0)	845 (73.2)	48 (70.6)	877 (73.0)	75 (72.8)	762 (73.2)	81 (66.9)
β blockers	555 (47.5)	22 (40.0)	541 (46.8)	36 (52.9)	568 (47.3)	47 (45.6)	492 (47.3)	61 (50.4)
Statins	755 (64.6)	35 (63.6)	745 (64.5)	45 (66.2)	765 (63.6)	**76 (73.8) ^d^**	669 (64.3)	77 (63.6)
Laboratory values on admission, n (%)								
Total cholesterol, mmol/L	5.26 ± 1.01	5.26 ± 0.96	5.27 ± 1.01	5.17 ± 0.91	5.27 ± 1.01	5.12 ± 0.91	5.25 ± 1.02	5.39 ± 0.96
Triglycerides, mmol/L	1.97 ± 0.98	2.06 ± 1.39	1.96 ± 1.01	1.84 ± 0.80	1.97 ± 0.98	1.94 ± 1.10	1.98 ± 1.00	1.94 ± 0.94
Family history of CVD, n (%)	542 (46.4)	28 (50.9)	537 (46.5)	33 (48.5)	558 (46.4)	48 (46.6)	486 (46.7)	55 (45.5)

ACB—Aortocoronary bypass; ACEIs—angiotensin-converting enzyme inhibitors; BMI—body mass index; CVD—cardiovascular disease; OACs—oral anticoagulants; PCI—Percutaneous coronary intervention; SD—standard deviation; * according to univariate Cox regression analysis; ** according to univariate logistic regression analysis; ^a^ *p* < 0.001; ^b^ *p* < 0.01; ^c^ *p* < 0.05; ^d^ *p* ≤ 0.10; statistically significant differences are bold.

**Table 2:** Characteristics of carotid disease, operative data and hospital discharge therapy according to the occurrence of myocardial infarction, stroke, death and restenosis as the late adverse outcomes of carotid endarterectomy.

Variable	Myocardial Infarction *		Stroke *		Death *		Restenosis **	
	NO (n = 1168)	YES (n = 55)	NO (n = 1155)	YES (n = 68)	NO (n = 1202)	YES (n = 103)	NO (n = 1041)	YES (n = 121)
Characteristics of carotid disease, n (%)								
Symptomatic	406 (34.8)	22 (40.0)	403 (34.9)	25 (36.8)	421 (35.0)	40 (38.8)	364 (35.0)	41 (33.9)
Complicated plaque	177 (15.2)	11 (20.0)	181 (15.7)	7 (10.3)	186 (15.5)	15 (14.7)	157 (15.1)	23 (19.0)
Ipsilateral stenosis > 70%	987 (84.5)	45 (81.8)	971 (84.1)	61 (89.7)			886 (85.1)	96 (79.3)
Contralateral stenosis > 70%	254 (21.7)	17 (30.1)	252 (21.8)	19 (27.9)	261 (21.7)	25 (24.3)	228 (21.9)	25 (20.6)
Operative data, n (%):								
Urgent endarterectomy	13 (1.1)	1 (1.8)	13 (1.1)	1 (1.5)	14 (1.2)	0 (0.0)	10 (1.0)	**4 (3.3) ^b^**
Clamp duration: <10 min 10–15 min >15 min	225 (19.5) 733 (63.5) 197 (17.1)	10 (18.2) 34 (61.8) 11 (20.0)	222 (19.4) 726 (63.5) 195 (17.1)	13 (19.4) 41 (61.2) 13 (19.4)	225 (18.9) 768 (64.5) 197 (16.6)	17 (16.7) 61 (59.8) 24 (23.5)	198 (19.2) 655 (63.6) 177 (17.2)	24 (20.0) 81 (67.5) 15 (12.5)
Hospital discharge therapy, n (%):								
Aspirin	1137 (97.3)	53 (96.4)	1126 (97.5)	**64 (94.1) ^c^**	1170 (97.3)	100 (97.1)	1011 (97.1)	119 (98.3)
Clopidogrel	808 (69.2)	41 (74.5)	804 (69.6)	45 (66.2)	836 (69.6)	78 (75.7)	728 (69.9)	76 (62.8)
OACs	45 (3.9)	**6 (10.9) ^b^**	47 (4.1)	4 (5.9)	46 (3.8)	**11 (10.7) ^a^**	40 (3.8)	5 (4.1)
ACEIs	919 (78.7)	42 (76.4)	910 (78.8)	51 (75.0)	948 (78.9)	80 (77.7)	816 (78.4)	95 (78.5)
β blockers	643 (55.1)	32 (58.2)	636 (55.1)	39 (57.4)	661 (55.0)	64 (62.1)	571 (54.9)	69 (57.0)
Statins	1132 (96.9)	54 (98.1)	1121 (97.0)	65 (95.5)	1167 (98.1)	100 (97.1)	1008 (96.8)	119 (98.3)

ACEIs—angiotensin-converting enzyme inhibitors; OACs—oral anticoagulants; * according to univariate Cox regression analysis; ** according to univariate logistic regression analysis; ^a^ *p* < 0.01; ^b^ *p* < 0.05; ^c^ *p* ≤ 0.10; statistically significant differences are bold.

**Table 3:** Results of crude, adjusted, and multiple imputed regression models for myocardial infarction, stroke, death and restenosis as the late adverse outcomes of CEA.

Variables	Myocardial Infarction (n = 55) 2nd Step HR (95% CI) *	Myocardial Infarction—After Adjustment HR (95% CI)	Multiple Imputation *** HR (95% CI)	Stroke (n = 68) 1st Step HR (95% CI) *	Stroke—After Adjustment HR (95% CI)	Multiple Imputation *** HR (95% CI)	Death (n = 103) Step 3rd HR (95% CI) *	Death—After Adjustment HR (95% CI)	Multiple Imputation *** HR (95% CI)	Restenosis (n = 121) 2nd Step OR (95% CI) **	Restenosis—After Adjustment HR (95% CI)	Multiple Imputation *** HR (95% CI)
Age	**1.08** **(1.05–1.11)**	**1.08** **(1.05–1.11)**	**1.08** **(1.05–1.11)**	**1.04** **(1.01–1.08)**	**1.04** **(1.01–1.08)**	**1.04** **(1.01–1.08)**	**1.17** **(1.12–1.21)**	**1.17** **(1.12–1.21)**	**1.13** **(1.09–1.16)**	/	/	/
Gender	**/**	**/**	**/**	**/**	**/**	**/**	**/**	**/**	0.67 (0.43–1.04)	/	/	/
ACB	**1.70** **(1.02–2.84)**	1.63 (0.95–2.78)	1.54 (0.90–2.63)	/	/	/	/	/	/	/	/	/
PAD	**1.83** **(1.20–2.80)**	**1.81** **(1.17–2.78)**	**1.80** **(1.17–2.78)**	/	/	/	/	/	/	/	/	/
Hyperlipidemia	/	/	/	/	/	/	/	**/**	**/**	**3.13** **(1.12–8.73)**	**3.52** **(1.27–9.76)**	**3.20** **(1.47–6.94)**
Urgent endarterectomy	/	/	/	/	/	/	/	**/**	**/**	**3.87** **(1.14–13.15)**	**3.51** **(1.06–11.65)**	**/**
Aspirin in hospital discharge therapy	/	/	/	0.42 (0.15–1.14)	0.43 (0.15–1.18)	0.40 (0.14–1.11)	/	**/**	**/**	**/**	**/**	**/**
OACs in hospital discharge therapy	/	**/**	**1.97** **(1.02–3.80)**	/	**/**	**/**	**2.16** **(1.15–4.08)**	2.20 (0.94–5.11)	**2.10** **(1.11–3.99)**	/	/	/
Diabetes Mellitus	**1.60** **(1.11–2.29)**	**1.60** **(1.11–2.29)**	**1.60** **(1.12–2.30)**	**1.66** **(1.02–2.70)**	**1.68** **(1.03–2.72)**	**1.64** **(1.01–2.66)**	**1.94** **(1.17–3.21)**	**1.94** **(1.17–3.21)**	**1.79** **(1.21–2.66)**	**1.75** **(1.19–2.58)**	**1.78** **(1.21–2.62)**	**1.47** **(1.06–2.02)**
Hypertension	/	/	/	/	**/**	**/**	**/**	/	/	2.34 (0.71–7.73)	/	/

ACB—Aortocoronary bypass; OACs—oral anticoagulants; PAD—peripheral arterial disease; * according to multivariate Cox regression analysis; ** according to multivariate logistic regression analysis; *** multiple imputation was assessed across 5 datasets using Rubin`s rules and pooled results of the analysis are presented. MI adjusted (both before and after multiple imputation) for all preoperative therapy (aspirin, clopidogrel, OACs, ACEIs, β blockers, statins) and cardiovascular comorbidities (except ACB, PAD and chronic heart failure); stroke adjusted for all preoperative therapy (aspirin, clopidogrel, OACs, ACEIs, β blockers, statins) and cardiovascular comorbidities (prior MI, ACB, PCI, PAD, CHF, AAA); death adjusted for all preoperative therapy (aspirin, clopidogrel, OACs, ACEIs, β blockers, statins) and cardiovascular comorbidities (prior MI, ACB, PCI, PAD, CHF, AAA); restenosis adjusted for all preoperative therapy (aspirin, clopidogrel, OACs, ACEIs, β blockers, statins) and cardiovascular comorbidities (prior MI, ACB, PCI, PAD, CHF, AAA); statistically significant differences are bold.

## Data Availability

The raw data supporting the conclusions of this article will be made available by the authors on request.

## Supplementary Materials

The following supporting information can be downloaded at: https://www.mdpi.com/article/10.3390/medicina62030593/s1, Table S1: Demographic characteristics, BMI, smoking, personal history, therapy, laboratory values on admission and family history of cardiovascular diseases in followed and non-followed patients; Table S2: Characteristics of carotid disease, operative data and hospital discharge therapy in followed and non-followed patients; Table S3: Step 1^st^ of adjusted regression models for myocardial infarction, stroke, death and restenosis)

## References

[1] Dorigo W., Pulli R., Pratesi G., Fargion A., Marek J., Innocenti A.A., Pratesi C. (2011). Early and long-term results of carotid endarterectomy in diabetic patients. J. Vasc. Surg..

[2] Pulli R., Dorigo W., Barbanti E., Azas L., Pratesi G., Innocenti A.A., Pratesi C. (2005). Does the high risk patient for carotid endarterectomy really exist?. Am. J. Surg..

[3] Go C., Avgerinos E.D., Chaer R.A., Ling J., Wazen J., Marone L., Fish L., Makaroun M.S. (2015). Long-Term Clinical Outcomes and Cardiovascular Events after Carotid Endarterectomy. Ann. Vasc. Surg..

[4] Vilariño-Rico J., Pita-Fernández S., Segura-Iglesias R.J. (2015). Clinical Predictors of Major Adverse Cardiovascular Events during Long-Term Follow-Up after Carotid Endarterectomy. Ann. Vasc. Surg..

[5] Alves-Ferreira J., Rocha-Neves J., Dias-Neto M., Braga S.F. (2019). Poor long-term outcomes after carotid endarterectomy: A retrospective analysis of two Portuguese centers. Scand. Cardiovasc. J..

[6] Mazzaccaro D., Modafferi A., Malacrida G., Nano G. (2019). Assessment of long-term survival and stroke after carotid endarterectomy and carotid stenting in patients older than 80 years. J. Vasc. Surg..

[7] Gajin P., Radak D.J., Tanaskovic S., Babic S., Nenezic D. (2014). Urgent carotid endarterectomy in patients with acute neurological ischemic events within six hours after symptoms onset. Vascular.

[8] North B.J., Sinclair D.A. (2012). The intersection between aging and cardiovascular disease. Circ. Res..

[9] Paneni F., Cañestro C.D., Libby P., Lüscher T.F., Camici G.G. (2017). The Aging Cardiovascular System Understanding It at the Cellular and Clinical Levels. J. Am. Coll. Cardiol..

[10] Kannel W.B., McGee D.L. (1979). Diabetes and cardiovascular disease. The Framingham study. JAMA.

[11] Blecha M., DeJong M., Carlson K. (2022). Risk factors for mortality within 5 years of carotid endarterectomy for asymptomatic stenosis. J. Vasc. Surg..

[12] Paraskevas K.I., Gloviczki P. (2020). Prognostic factors of long-term survival to guide selection of asymptomatic patients for carotid endarterectomy. Int. Angiol..

[13] Rizwan M., Aridi H.D., Dang T., Alshwaily W., Nejim B., Malas M.B. (2019). Long-Term Outcomes of Carotid Endarterectomy and Carotid Artery Stenting When Performed by a Single Vascular Surgeon. Vasc. Endovascular Surg..

[14] Kang J., Conrad M.F., Patel V.I., Mukhopadhyay S., Garg A., Cambria M.R., LaMuraglia G.M., Cambria R.P. (2014). Clinical and anatomic outcomes after carotid endarterectomy. J. Vasc. Surg..

[15] Kragsterman B., Björck M., Lindbäck J., Bergqvist D., Pärsson H., Swedish Vascular Registry (Swedvasc) (2006). Long-Term Survival After Carotid Endarterectomy for Asymptomatic Stenosis. Stroke.

[16] Imahori Y., Mathiesen E.B., Morgan K.E., Frost C., Hughes A.D., Hopstock L.A., Johnsen S.H., Emaus N., Leon D.A. (2020). The association between anthropometric measures of adiposity and the progression of carotid atherosclerosis. BMC Cardiovasc. Disord..

[17] Ucci A., de Troia A., D’Ospina R.M., Pedrazzi G., Nabulsi B., Azzarone M., Perini P., Massoni C.B., Rossi G., Freyrie A. (2023). Carotid endarterectomy in asymptomatic octogenarians: Outcomes at 30 days and 5 years. Vascular.

[18] Casana R., Malloggi C., Odero A., Tolva V., Bulbulia R., Halliday A., Silani V. (2018). Is diabetes a marker of higher risk after carotid revascularization? Experience from a single centre. Diab Vasc. Dis. Res..

[19] Katsiki N., Mikhailidis D.P. (2020). Diabetes and carotid artery disease: A narrative review. Ann. Transl. Med..

[20] Pothof A.B., O’Donnell T.F.X., Swerdlow N.J., Liang P., Li C., Varkevisser R.R.B., de Borst G.J., Schermerhorn M.L. (2019). Risk of insulin-dependent diabetes mellitus in patients undergoing carotid endarterectomy. J. Vasc. Surg..

[21] Jeong M.-J., Kwon H., Jung C.H., Kwon S.U., Kim M.-J., Han Y., Kwon T.-W., Cho Y.-P. (2019). Comparison of outcomes after carotid endarterectomy between type 2 diabetic and non-diabetic patients with significant carotid stenosis. Cardiovasc. Diabetol..

[22] Dimic A., Markovic M., Vasic D., Dragas M., Zlatanovic P., Mitrovic A., Davidovic L. (2019). Impact of diabetes mellitus on early outcome of carotid endarterectomy. Vasa.

[23] Macharzina R.R., Müller C., Vogt M., Messé S.R., Vach W., Winker T., Weinbeck M., Siepe M., Czerny M., Neumann F.-J. (2020). The SAPPHIRE criteria, history of myocardial infarction and diabetes predict adverse outcomes following carotid endarterectomy similar to stenting. Clin. Res. Cardiol.

[24] Ballotta E., Meneghetti G., Manara R., Baracchini C. (2007). Long-term survival and stroke-free survival after eversion carotid endarterectomy for asymptomatic severe carotid stenosis. J. Vasc. Surg..

[25] LaMuraglia G.M., Brewster D.C., Moncure A.C., Dorer D.J., Stoner M.C., Trehan S.K., Drummond E.C., Abbott W.M., Cambria R.P. (2004). Carotid Endarterectomy at the Millennium: What Interventional Therapy Must Match. Ann. Surg..

[26] Hoke M., Schillinger M., Minar E., Goliasch G., Binder C.J., Mayer F.J. (2019). Carotid ultrasound investigation as a prognostic tool for patients with diabetes mellitus. Cardiovasc. Diabetol..

[27] Hussain M.A., Bin-Ayeed S.A., Saeed O.Q., Verma S., Al-Omran M. (2016). Impact of diabetes on carotid artery revascularization. J. Vasc. Surg..

[28] Zabala A., Gottsäter A., Lind M., Svensson A.M., Eliasson B., Bertilsson R., Ekelund J., Nyström T., Jonsson M. (2021). Early and long term prognosis in patients with and without type 2 diabetes after carotid intervention: A Swedish nationwide propensity score matched cohort study. Cardiovasc. Diabetol..

[29] Zabala A., Gottsäter A., Lind M., Eliasson B., Bertilsson R., Ekelund J., Jonsson M., Nyström T. (2023). Glycemic control and outcome after carotid intervention in patients with T2D: A Swedish nationwide cohort study. Diab Vasc. Dis. Res..

[30] Texakalidis P., Tzoumas A., Giannopoulos S., Jonnalagadda A.K., Jabbour P., Rangel-Castilla L., Machinis T., Rivet D.J., Reavey-Cantwell J. (2019). Risk Factors for Restenosis After Carotid Revascularization: A Meta-Analysis of Hazard Ratios. World Neurosurg..

[31] Lal B.K., Beach K.W., Roubin G.S., Lutsep H.L., Moore W.S., Malas M.B., Chiu D., Gonzales N.R., Burke J.L., Rinaldi M. (2012). Restenosis after carotid artery stenting and endarterectomy: A secondary analysis of CREST, a randomised controlled trial. Lancet Neurol..

[32] Fluri F., Hatz F., Voss B., Lyrer P.A., Engelter S.T. (2010). Restenosis after carotid endarterectomy: Significance of newly acquired risk factors. Eur. J. Neurol..

[33] Reina-Gutiérrez T., Serrano-Hernando F.J., Sánchez-Hervás L., Ponce A., Vega de Ceniga M., Martín A. (2005). Reccurent carotid artery stenosis following endarterectomy: Natural history and risk factors. Eur. J. Vasc. Endovasc. Surg..

[34] van Lammeren G.W., Catanzariti L.M., Peelen L.M., de Vries J.-P.P.M., de Kleijn D.P.V., Moll F.L., Pasterkamp G., Bots M.L. (2012). Clinical prediction rule to estimate the absolute 3-year risk of major cardiovascular events after carotid endarterectomy. Stroke.

[35] Ogata T., Inoue T., Okada Y. (2014). Outcome of 312 Japanese patients with carotid endarterectomy and factors associated with cardiovascular events--a single-center study in Japan. J. Stroke Cerebrovasc. Dis..

[36] Petrovic J., Ilijevski N., Sagic D., Antonic Z., Tanaskovic S. (2023). Risk Factors for Carotid Restenosis in Patients After Eversion Endarterectomy vs Stenting: A Single-Center Experience. Angiology.

[37] Garzon-Muvdi T., Yang W., Rong X., Caplan J.M., Ye X., Colby G.P., Coon A.L., Tamargo R.J., Huang J. (2016). Restenosis After Carotid Endarterectomy: Insight Into Risk Factors and Modification of Postoperative Management. World Neurosurg..

[38] Volteas N., Labropoulos N., Leon M., Kalodiki E., Chan P., Nicolaides A.N. (1994). Risk factors associated with recurrent carotid stenosis. Int. Angiol..

[39] Mughal M.M., Khan M.K., DeMarco J.K., Majid A., Shamoun F., Abela G.S. (2011). Symptomatic and asymptomatic carotid artery plaque. Expert. Rev. Cardiovasc. Ther..

[40] Strömberg S., Gelin J., Osterberg T., Bergström G.M.L., Karlström L., Osterberg K., Swedish Vascular Registry (Swedvasc) Steering Committee (2012). Very urgent carotid endarterectomy confers increased procedural risk. Stroke.

[41] Nordanstig A., Rosengren L., Strömberg S., Österberg K., Karlsson L., Bergström G., Fekete Z., Jood K. (2017). Editor’s Choice—Very Urgent Carotid Endarterectomy is Associated with an Increased Procedural Risk: The Carotid Alarm Study. Eur. J. Vasc. Endovasc. Surg..

[42] Chen P., Lazar A., Ding J., Siracuse J.J., Patel V.I., Morrissey N.J. (2023). Insurance status is associated with urgent carotid endarterectomy and worse postoperative outcomes. J. Vasc. Surg..

[43] Cui C.L., Yei K.S., Ramachandran M., Mwinyogle A., Malas M.B. (2022). In-hospital complications and long-term outcomes associated with timing of carotid endarterectomy. J. Vasc. Surg..

[44] De Blasis S., Pulli R., Di Domenico R., Nesi M., Nencini P., Fargion A.T., Pratesi C., Dorigo W. (2023). Elective or Urgent Carotid Endarterectomy in Symptomatic Patients: Analysis Based on the Type and Timing of Neurological Symptoms. Ann. Vasc. Surg..

[45] Bonati L.H., Gregson J., Dobson J., McCabe D.J.H., Nederkoorn P.J., van der Worp H.B., de Borst G.J., Richards T., Cleveland T., Müller M.D. (2018). Restenosis and risk of stroke after stenting or endarterectomy for symptomatic carotid stenosis in the International Carotid Stenting Study (ICSS): Secondary analysis of a randomised trial. Lancet Neurol..

[46] Kumar R., Batchelder A., Saratzis A., AbuRahma A.F., Ringleb P., Lal B.K., Mas J.L., Steinbauer M., Naylor A.R. (2017). Restenosis after Carotid Interventions and Its Relationship with Recurrent Ipsilateral Stroke: A Systematic Review and Meta-analysis. Eur. J. Vasc. Endovasc. Surg..

[47] Naylor R., Rantner B., Ancetti S., de Borst G.J., De Carlo M., Halliday A., Kakkos S.K., Markus H.S., McCabe D.J., Sillesen H. (2023). Editor’s Choice—European Society for Vascular Surgery (ESVS) 2023 Clinical Practice Guidelines on the Management of Atherosclerotic Carotid and Vertebral Artery Disease. Eur. J. Vasc. Endovasc. Surg..

