# Supplementary material for: Predictors of Late Adverse Outcomes After Carotid Endarterectomy
Source: Medicina (Kaunas). 2026 Mar 21;62(3):593. doi: 10.3390/medicina62030593 (PMC13028278; doi:10.3390/medicina62030593)
Supplement: Supplementary file 1 [file medicina-62-00593-s001.zip › Supplement S1.pdf]

Table S1. Demographic characteristics, BMI, smoking, personal history, therapy, laboratory values on admission and family history of cardiovascular diseases in followed and non-followed patients

**Variable**	Followed patients n=1223 (76.6%)	Non-followed patients n=374 (23.4%)	p value*
Age, y, mean ± SD	68.01±7.7	70.23±7.8	**<0.001**
Gender – Male, n (%)	726 (59.4)	234 (62.7)	0.230
BMI: underweight<br>normalweight<br>overweight<br>obese	0 (0.0)<br>413 (33.8)<br>583 (47.7)<br>220 (18.0)	0 (0.0)<br>137 (36.7)<br>166 (44.5)<br>66 (17.7)	0.282
Smoking, n (%)	572 (46.8)	182 (48.8)	0.464
Personal history, n (%):			
Myocardial infarction	88 (7.2)	28 (7.5)	0.849
PCI	78 (6.4)	26 (7.0)	0.694
ACB	101 (8.3)	28 (7.5)	0.632
Chronic heart failure	21 (1.7)	5 (1.3)	0.611
Peripheral arterial disease	191 (15.6)	60 (16.1)	0.843
Aneurysmatic disease	40 (3.3)	6 (1.6)	0.092
Hyperlipidemia	1098 (89.8)	326 (87.4)	0.200
Hypertension	1145 (93.6)	347 (93.0)	0.694
Diabetes mellitus	372 (30.4)	122 (32.7)	0.420
Therapy before admission, n (%):			
Aspirin	1006 (82.3)	293 (78.6)	0.113
Clopidogrel	335 (27.4)	105 (28.2)	0.796
OACs	58 (4.7)	15 (4.0)	0.553
ACEIs	893 (73.0)	261 (70.0)	0.262
β blockers	577 (47.2)	164 (44.0)	0.259
Statins	790 (64.6)	238 (63.8)	0.735
Laboratory values on admission, n (%)			
Cholesterol ≥5.2 mmol/L	583 (47.7)	181 (48.5)	0.826
Triglycerides ≥ 1.7 mmol/L	649 (53.1)	194 (52.0)	0.593
Family history of CVD, n (%)	570 (46.6)	161 (43.2)	0.227

ACB – Aortocoronary bypass; ACEIs - angiotensin-converting enzyme inhibitors; BMI – body mass index; CVD – cardiovascular disease; OACs - oral anticoagulants; PCI – Percutaneous coronary intervention; SD – standard deviation;

*For significance level of 0.05 according to Students-t test and Chi-square test

Table S2. Characteristics of carotid disease, operative data and hospital discharge therapy in followed and non-followed patients

Variable	Followed patients n=1223 (76.6%)	Non-followed patients n=374 (23.4%)	P value*
Characteristics of carotid disease, n (%):			
Symptomatic	428 (35.0)	134 (35.9)	0.768
Complicated plaque	188 (15.4)	55 (14.7)	0.760
Ipsilateral stenosis			0.957
50-69%	186 (15.2)	57 (15.3)	
70-89%	671 (54.9)	203 (54.4)	
90-99%	361 (29.5)	111 (29.8)	
Contralateral stenosis			0.347
50-69%	206 (16.8)	49 (13.1)	
70-89%	118 (9.6)	41 (11.0)	
90-99%	51 (4.2)	17 (4.6)	
100%	102 (8.3)	31 (8.3)	
Operative data, n (%):			
Urgent endarterectomy	14 (1.1)	2 (0.5)	0.300
Clamp duration:<br><10 min<br>10 – 15 min<br>>15 min	 235 (19.2)<br>767 (62.7)<br>208 (17.0)	 65 (17.4)<br>236 (63.3)<br>70 (18.8)	0.606
Hospital discharge therapy, n (%):			
Aspirin	1190 (97.3)	356 (95.4)	0.978
Clopidogrel	849 (69.4)	261 (70.0)	0.471
OACs	51 (4.2)	18 (4.8)	0.545
ACEIs	961 (78.6)	286 (76.7)	0.878
β blockers	675 (55.2)	203 (54.4)	0.967
Statins	1186 (97.0)	352 (94.4)	0.452

ACEIs – angiotensin-converting enzyme inhibitors; OACs - oral anticoagulants;

*For significance level of 0.05 according to Students-t test and Chi-square test
