# Supplementary material for: Predictors of Late Adverse Outcomes After Carotid Endarterectomy
Source: Medicina (Kaunas). 2026 Mar 21;62(3):593. doi: 10.3390/medicina62030593 (PMC13028278; doi:10.3390/medicina62030593)
Supplement: Supplementary file 1 [file medicina-62-00593-s001.zip › Supplement S2.pdf]

Supplement S2. Step 1st of adjusted regression models for myocardial infarction, stroke, death and restenosis

Variables	Myocardial infarction (n=55)<br>Step 1st<br>HR (95% CI)	Stroke (n=68)<br>Step 1st<br>HR (95% CI)	Death (n=103)<br>Step 1st<br>HR (95% CI)	Restenosis (n=121)<br>Step 1st<br>OR (95% CI)
Age	**1.08 (1.05-1.11)**[a]	**1.04 (1.01-1.08)**[c]	**1.17 (1.12-1.21)**[a]	/
Gender	/	/	0.64 (0.36-1.12)	/
ACB	1.67 (1.00-2.81)[a]	/	/	/
PAD	**1.83 (1.20-2.81)**[c]	/	/	/
Hyperlipoproteinemia	/	/	/	**3.13 (1.12-8.73)**[c]
Urgent endarterectomy	/	/	/	**3.88 (1.14-13.15)**[c]
OACs in hospital discharge therapy	1.23 (0.60-2.53)	/	**2.00 (0.85-4.69)**[c]	/
Aspirin in hospital discharge therapy		0.42 (0.15-1.14)		
Diabetes Mellitus	**1.60 (1.12-2.30)**	1.66 (1.02-2.70)	**1.96 (1.18-3.25)**[b]	**1.75 (1.19-2.58)**[b]
Hypertension	/	/	/	2.34 (0.71-7.73)

ACB – Aortocoronary bypass; OACs - oral anticoagulants; PAD – Peripheral arterial disease; CHF – chronic hearth failure

*According to multivariate Cox regression analysis; ** According to multivariate logistic regression analysis;

[a] $p<0.001$; [b] $p<0.01$; [c] $p<0.05$.

Myocardial infarction adjusted for all preoperative thepary and cardiovascular comorbidities (except ACB, PAD and chronic heart failure);

Stroke adjusted for all preoperative therapy and cardiovasculasr comorbidities (prior MI, ACB, PCI, PAD, CHF, AAA);

Death adjusted for all preoperative therapy and cardiovascular comorbidities (prior MI, ACB, PCI, PAD, CHF, AAA);

Restenosis adjusted for all preoperative therapy and cardiovascular comorbidities (prior MI, ACB, PCI, PAD, CHF, AAA)
